# Estimation of Transmission of COVID-19 in Simulated Nursing Homes With Frequent Testing and Immunity-Based Staffing

**DOI:** 10.1001/jamanetworkopen.2021.10071

**Published:** 2021-05-14

**Authors:** Inga Holmdahl, Rebecca Kahn, James A. Hay, Caroline O. Buckee, Michael J. Mina

**Affiliations:** 1 Center for Communicable Disease Dynamics, Department of Epidemiology, Harvard T.H. Chan School of Public Health, Boston, Massachusetts; 2 Department of Immunology and Infectious Diseases, Harvard T.H. Chan School of Public Health, Boston, Massachusetts; 3 Department of Pathology, Brigham and Women’s Hospital, Harvard Medical School, Boston, Massachusetts

## Abstract

**Importance:**

Nursing homes and other long-term care facilities have been disproportionately impacted by the COVID-19 pandemic. Strategies are urgently needed to reduce transmission in these high-risk populations.

**Objective:**

To evaluate COVID-19 transmission in nursing homes associated with contact-targeted interventions and testing.

**Design, Setting, and Participants:**

This decision analytical modeling study developed an agent-based susceptible–exposed–infectious (asymptomatic/symptomatic)–recovered model between July and September 2020 to examine SARS-CoV-2 transmission in nursing homes. Residents and staff of a simulated nursing home with 100 residents and 100 staff split among 3 shifts were modeled individually; residents were split into 2 cohorts based on COVID-19 diagnosis. Data were analyzed from September to October 2020.

**Exposures:**

In the resident cohorting intervention, residents who had recovered from COVID-19 were moved back from the COVID-19 (ie, infected with SARS-CoV-2) cohort to the non–COVID-19 (ie, susceptible and uninfected with SARS-CoV-2) cohort. In the immunity-based staffing intervention, staff who had recovered from COVID-19 were assumed to have protective immunity and were assigned to work in the non–COVID-19 cohort, while susceptible staff worked in the COVID-19 cohort and were assumed to have high levels of protection from personal protective equipment. These interventions aimed to reduce the fraction of people’s contacts that were presumed susceptible (and therefore potentially infected) and replaced them with recovered (immune) contacts. A secondary aim of was to evaluate cumulative incidence of SARS-CoV-2 infections associated with 2 types of screening tests (ie, rapid antigen testing and polymerase chain reaction [PCR] testing) conducted with varying frequency.

**Main Outcomes and Measures:**

Estimated cumulative incidence proportion of SARS-CoV-2 infection after 3 months.

**Results:**

Among the simulated cohort of 100 residents and 100 staff members, frequency and type of testing were associated with smaller outbreaks than the cohorting and staffing interventions. The testing strategy associated with the greatest estimated reduction in infections was daily antigen testing, which reduced the mean cumulative incidence proportion by 49% in absence of contact-targeted interventions. Under all screening testing strategies, the resident cohorting intervention and the immunity-based staffing intervention were associated with reducing the final estimated size of the outbreak among residents, with the immunity-based staffing intervention reducing it more (eg, by 19% in the absence of testing) than the resident cohorting intervention (eg, by 8% in the absence of testing). The estimated reduction in transmission associated with these interventions among staff varied by testing strategy and community prevalence.

**Conclusions and Relevance:**

These findings suggest that increasing the frequency of screening testing of all residents and staff, or even staff alone, in nursing homes may reduce outbreaks in this high-risk setting. Immunity-based staffing may further reduce spread at little or no additional cost and becomes particularly important when daily testing is not feasible.

## Key Points

**Question:**

What are the associations of cohorting, staffing, and testing interventions with COVID-19 transmission in nursing homes?

**Findings:**

In this decision analytical modeling study in a simulated nursing home with 100 residents and 100 staff, routine screening testing and strategies that prioritized pairing recovered staff and recovered residents with susceptible residents were associated with a reduction in transmission of COVID-19 in nursing homes.

**Meaning:**

These findings suggest that frequent testing and immunity-based staffing interventions may reduce transmission of SARS-CoV-2 in nursing homes and protect this vulnerable population.

## Teaser

This decision analytical modeling study examines the associations of cohorting, staffing, and testing interventions with COVID-19 transmission in nursing homes.

## Introduction

In the United States, nursing homes and other long term care facilities have been disproportionately affected by the COVID-19 pandemic.^1,2^ As of September 2020, 40% of COVID deaths nationwide were linked to nursing homes.^3^ Nursing home residents are at particularly high risk for severe disease and mortality owing to older age and high prevalence of underlying medical conditions. In addition, nursing homes have high risk of transmission because residents live in close quarters and have frequent close contact with staff.^4^ Furthermore, many facilities face understaffing, which may lead to even higher contact rates between staff and residents, and larger outbreaks.^5^

The ability to develop evidence-based responses to this crisis has been constrained by limited data and research in these facilities prior to the COVID-19 pandemic. SARS-CoV-2 infection control in many nursing homes has relied on isolating residents who were infected in a COVID-19 cohort, per recommendations.^5,6^ Long-term separation after recovery keeps the non–COVID-19 cohort highly susceptible, and thus has the potential unintended effect of preventing institutional herd immunity. Additionally, there is relatively little evidence about the potential of staffing strategies based on infection histories. Although guidelines require testing in nursing homes,^6^ the frequency and type of testing varies.

Despite guidelines on cohorting and testing, nursing homes have experienced large outbreaks.^7^ A primary driver may be presymptomatic and asymptomatic transmission, the latter of which occurs in an estimated 40% of SARS-CoV-2 infections.^8,9^ For this reason, public health screening testing (ie, testing individuals who are not symptomatic) is an important intervention to control transmission. In addition, control measures that reduce the frequency of contacts among those who are susceptible and those who are infected but not yet identified could reduce transmission. Owing to a lack of resources, it is critical to identify interventions that will most effectively control infection in these settings at minimal cost.

In this study, we use an agent-based model representing a nursing home to estimate the associations of a range of interventions with COVID-19 outbreak size. We focus on 2 contact-targeted interventions to group staff and residents based on their infection and immunity status and examine testing interventions that reflect currently available virological tests. Our proposed strategies involve colocating susceptible nursing home residents with staff and residents who have recovered and are assumed to be immune, and examining whether screening with rapid antigen testing outperforms polymerase chain reaction (PCR)–based testing strategies.

## Methods

### Model Structure

**Figure 1.**
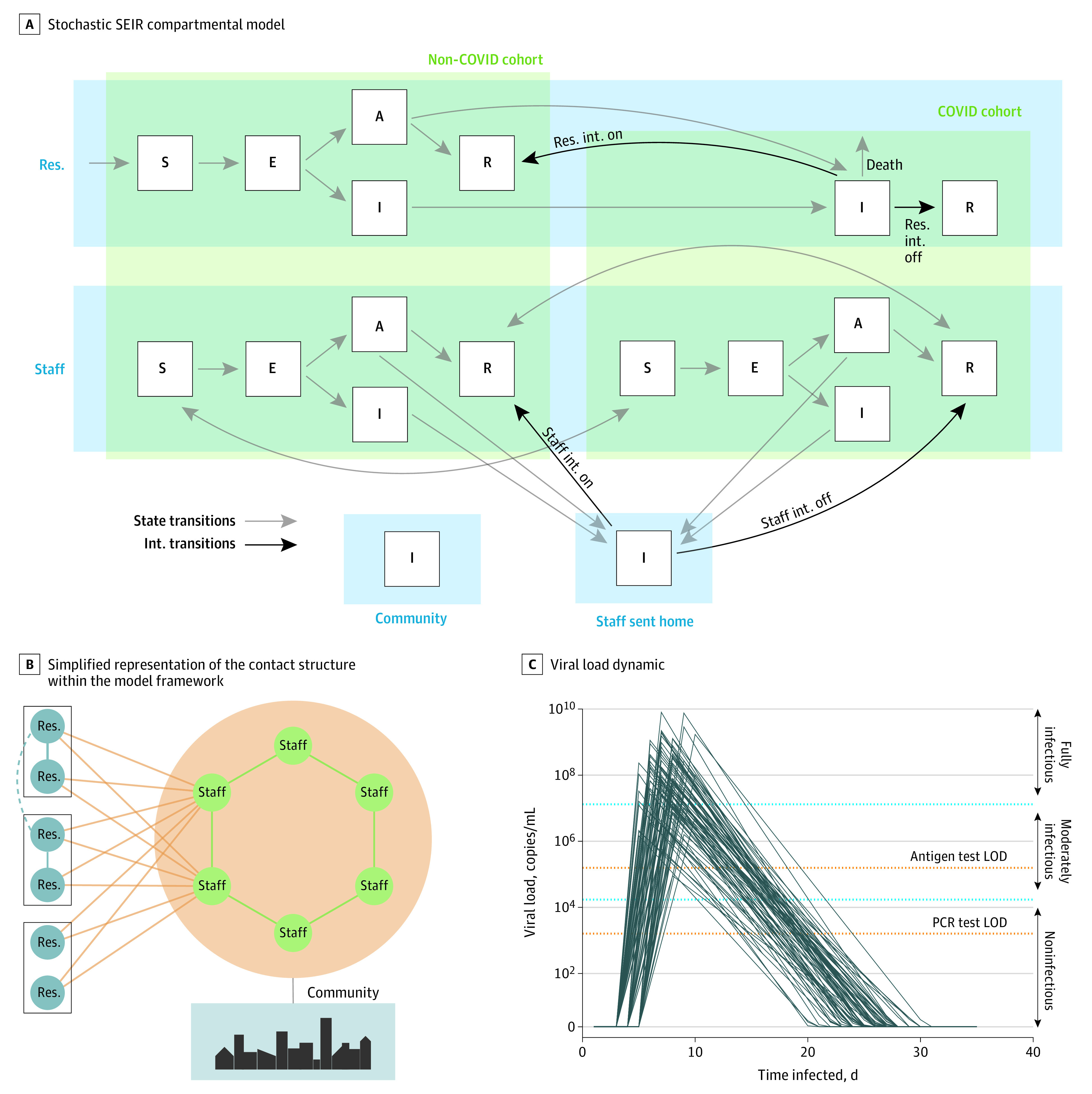
Susceptible–Exposed–Infectious (Asymptomatic/Symptomatic)–Recovered (SEIR) Model A, Flowchart of movement through the SEIR compartmental model. This model uses a cohorted framework wherein nursing home residents who become infected either symptomatically (I) or asymptomatically (A) with SARS-CoV-2 are moved out of the non–COVID-19 cohort and separated into a distinct COVID-19 cohort after showing symptoms or testing positive. R indicates individuals who have recovered from (and are assumed immune to) COVID-19; res., residents; and int., intervention. Black lines denote cohorting interventions and gray lines show transitions between infection states. B, There are 2 residents per room, and each interacts with 6 staff per day. Staff interact with 2 other staff each day and also have a daily risk of infection from the community. The dashed line indicates sensitivity analyses with additional limited contacts between residents (eFigure 7 in the Supplement). C, LOD indicates limit of detection; PCR, polymerase chain reaction.

This decision analytical modeling study did not require institutional review board review or approval because only simulated data are used. In this study, we developed a stochastic, agent-based, discrete-time susceptible–exposed–infectious (asymptomatic/symptomatic)–recovered model to examine SARS-CoV-2 transmission in nursing homes. We modeled daily time steps over 3 months, beginning with a fully susceptible population and 1 resident with SARS-CoV-2 exposure. Once identified, residents who were infected were separated into a distinct cohort where they have no interactions with residents who are susceptible or undiagnosed (Figure 1). Individuals with SARS-CoV-2 infection were either asymptomatic, in which case they could only be identified through testing, or symptomatic, in which case they were identified either by symptom onset or testing, whichever occurred first. When individuals developed symptoms, they were presumed positive and either isolated (residents) or sent home (staff) regardless of test turnaround time. Residents were assumed to have a lower probability of asymptomatic infection than staff owing to age and other risk factors (eTable 1 in the Supplement).

We used 2016 Centers for Medicare & Medicaid Services data to set the distribution for the length of stay for residents.^10^ We made the conservative assumption that new residents continually replaced those who died or were discharged, keeping capacity at 100% and leading to a constant inflow of susceptible residents. On entry into the nursing home, each resident was assigned to a 2-person room and remained in there unless identified as infected and moved to the COVID-19 cohort or discharged.

Staff were split between COVID-19 and non–COVID-19 cohorts in proportion to the number of residents in each. Staff who became infected were sent home and replaced by temporary workers while they recovered. Staff had a constant daily risk of infection from the community, which was set to reflect different levels of community prevalence (eTable 1 in the Supplement). We examined a range of values for the probability of infection. For our primary analysis, we used our highest value to understand intervention effectiveness in settings with intense community transmission, such as winter 2020 to 2021 in many parts of the US.^7,11^

Daily contacts were modeled between residents, staff, and between the 2 populations. We assumed transmission only occurred on contact and did not consider strictly airborne transmission. The frequency of contacts between residents and staff were based on rates in a Massachusetts network of nursing homes, as reported by R. Anglo, RN, MSN, MBA (Chelsea Jewish Life Care, verbal and email communication, June 10, 2020), which was consistent with analyses of nursing homes across the country, as reported by of M. Samore, MD (University of Utah, verbal and email communication, December 8, 2020). With the exception of roommates, who were modeled explicitly, we assumed homogeneous (ie, random) mixing. The daily risk of infection for each individual was binomial, with a probability determined by the mean infectiousness of the population they contacted multiplied by the number of contacts. At baseline, staff contacted 6 residents per day and 2 other staff. As nursing homes have been under strict restrictions during the pandemic,^12^ each resident only interacted with their roommate and staff but not other residents. Each of these types of contact (staff-resident, staff-staff, and resident-resident) was weighted to represent different contact intensities. These did not incorporate proximity and duration of contact explicitly, but aimed to capture heterogeneity in transmission risk. The lowest intensity was between staff, with a baseline probability of transmission per contact of 0.02. Owing to closer contact necessitated by provision of care, staff-resident contacts had a 3-fold higher risk of transmission (0.06) than baseline. Roommates had the highest risk of transmission, which was 10-fold higher (0.20) than baseline.

### Viral Load and Infectiousness

For each individual, viral load followed a tent-function approximating current data on viral shedding (Figure 1C).^13,14^ Following the latent period, viral load increased rapidly for 2 to 5 days before peaking, then immediately declined. For individuals who were symptomatic, symptom onset occurred after 2 days of increasing viral load, resulting in an incubation period of 5 to 7 days.^15,16^ We made the conservative assumption of the same viral load distributions for asymptomatic and symptomatic infections.^17^ The peak viral load was drawn from a normal distribution on the log scale.^18^ The duration of detectable viral load was normally distributed and could be longer than the infectious period.^19^ The relationship between viral load and infectiousness is not fully understood.^20^ In this model, we based infectiousness categorically on viral load: not infectious, moderately infectious, and fully infectious (Figure 1C; eTable 1 in the Supplement). We assumed the sensitivity of the tests only depended on viral dynamics and did not incorporate other factors, such as variations in sample quality. We set the probability of infection given an infectious contact so as to observe a plausible basic reproductive number, *R_0_*, in the absence of interventions (eTable 1 in the Supplement).^21^

### Simulated Interventions

We evaluated 2 interventions targeting contact patterns, which started 3 weeks into the outbreak (Table 1). Because we assumed that the contacts in nursing homes are necessary, our interventions did not reduce contacts but rather changed with whom those contacts occurred. Whenever possible, these interventions reduced the proportion of people’s contacts that were presumed susceptible (and therefore potentially infected) and replaced them with recovered (immune) contacts. We considered baseline as a state in which residents who were identified as infected move into isolation in a separate COVID-19 cohort and were not moved back into the non–COVID-19 cohort once they recovered (R. Anglo, RN, MSN, MBA, verbal and email communication, June 10, 2020). Under our resident cohorting intervention, residents were moved back to the non–COVID-19 cohort after recovery and were prioritized as roommates for new residents. The immunity-based staffing intervention prioritized placing recovered staff, who we assumed were immune, in the non–COVID-19 cohort, leaving susceptible staff to work in the COVID-19 cohort. Importantly, we assumed staff working in the COVID-19 cohort were provided adequate personal protective equipment (PPE) that reduced infection risk.

**Table 1.** Testing Interventions

Testing intervention	Staff frequency	Resident frequency	Limit of detection	Turnaround time, d
None	NA	NA	10^5^	<1
Weekly antigen	Weekly	Weekly	10^5^	<1
Weekly antigen, staff only	Weekly	NA	10^5^	<1
2.3 times/wk antigen	2.3 times/wk	2.3 times/wk	10^5^	<1
2.3 times/wk antigen, staff only	2.3 times/wk	NA	10^5^	<1
Daily antigen	Daily	Daily	10^5^	<1
Daily antigen, staff only	Daily	NA	10^5^	<1
Weekly PCR	Weekly	Weekly	10^3^	2
2.3 times/wk PCR	2.3 times/wk	2.3 times/wk	10^3^	2
Daily PCR	Daily	Daily	10^3^	2

Abbreviation: PCR, polymerase chain reaction.

In the testing interventions, which started immediately, we evaluated 2 types of screening tests—rapid antigen and PCR—at different frequencies, targeting either staff only or the entire nursing home (Table 1). In all scenarios, we assumed that symptom-based testing was conducted to confirm infection. Individuals who were waiting on test results were assumed to behave as normal unless they experienced symptoms before receiving results, in which case residents were moved to the COVID-19 cohort and staff were sent home. Based on the current parameters of these tests, antigen testing was assumed to have a higher limit of detection (LOD) and consequently lower sensitivity than PCR but returned results immediately, whereas PCR had a 2-day delay (eTable 1 and eTable 2 in the Supplement). Because the viral load cutoff for infectiousness was higher than the PCR test LOD, PCR test sensitivity to detect infectiousness was 100%. We estimated antigen test sensitivity for infectiousness by calculating the mean proportion of infectious days detectable by antigen tests. In the baseline simulations, we assumed perfect specificity, such as through the use of rapid orthogonal molecular– or antigen-based confirmatory tests. In sensitivity analyses, we relaxed this assumption among staff and assumed a 2-day delay in confirmatory testing, during which time staff were replaced by temporary staff. We further explored variations in the sensitivity of the antigen tests and the turnaround time of the PCR tests (eTable 1 and eTable 2 in the Supplement). To evaluate effectiveness associated with each intervention, we compared the mean and distribution of the cumulative incidence proportion after 3 months from 100 stochastic simulations.

We conducted additional sensitivity analyses of our assumptions about epidemic dynamics by varying the force of infection, β (eTable 1 in the Supplement). We also examined the impact associated with the relationship between viral load and infectiousness by removing infectiousness categories and making anyone who was in the infectious compartments equally infectious. We varied the ratio of staff to residents and resulting contact patterns, as well as increased contact rates between residents. To understand the importance of PPE, we conducted simulations with much lower PPE efficacy, which could also reflect imperfect adherence or limited supply.

All analyses were conducted in R statistical software version 3.6.3 (R Project for Statistical Computing). The code used for this study is available online.^22^ Data were analyzed from September to October 2020.

## Results

In a simulated nursing home with 100 residents and 100 staff, the estimated mean (SD) cumulative incidence proportion across 100 simulations after 3 months in the absence of testing or cohorting interventions was 0.41 (0.06) (Table 2). The mean (SD) cumulative incidence was 0.26 (0.06) among residents and 0.50 (0.06) among staff. We define estimated effectiveness as the reduction in mean cumulative incidence relative to the mean of a baseline scenario. In the absence of testing, the estimated effectiveness of the resident cohorting intervention compared with no intervention was 8% for residents, 4% for staff (excluding community infections), and 2% overall (including community introductions). The estimated effectiveness associated with this intervention was lower when testing frequency increased, and led to no change in overall cumulative incidence proportion in the highest frequency testing scenarios. However, the resident cohorting intervention reduced the total number of residents in the COVID-19 cohort, therefore also reducing the number of staff working in the COVID-19 cohort each day (eFigure 1 in the Supplement); this has implications for the amount of PPE required.

**Table 2.** Mean Cumulative Incidence Proportion Over 3 Months

Test strategy	Mean (SD)			
	None	Resident intervention	Staff intervention	Both interventions
**Residents**				
None	0.26 (0.06)	0.24 (0.06)	0.21 (0.05)	0.23 (0.05)
Weekly antigen	0.12 (0.04)	0.12 (0.03)	0.12 (0.03)	0.12 (0.03)
Weekly antigen, staff only	0.14 (0.05)	0.13 (0.04)	0.12 (0.04)	0.12 (0.03)
2.3 times/wk antigen	0.08 (0.03)	0.08 (0.02)	0.07 (0.02)	0.07 (0.02)
2.3 times/wk antigen, staff only	0.09 (0.03)	0.09 (0.03)	0.08 (0.03)	0.08 (0.03)
Daily antigen	0.04 (0.02)	0.05 (0.02)	0.04 (0.02)	0.04 (0.02)
Daily antigen, staff only	0.05 (0.02)	0.05 (0.02)	0.05 (0.02)	0.05 (0.02)
Weekly PCR	0.17 (0.06)	0.15 (0.04)	0.14 (0.04)	0.13 (0.04)
2.3 times/wk PCR	0.11 (0.03)	0.10 (0.03)	0.10 (0.03)	0.10 (0.03)
Daily PCR	0.07 (0.02)	0.07 (0.02)	0.07 (0.02)	0.07 (0.02)
**Staff (nursing home origin of infection)**				
None	0.50 (0.06)	0.48 (0.05)	0.45 (0.05)	0.47 (0.06)
Weekly antigen	0.34 (0.04)	0.35 (0.04)	0.34 (0.03)	0.34 (0.04)
Weekly antigen, staff only	0.38 (0.04)	0.37 (0.04)	0.36 (0.04)	0.36 (0.03)
2.3 times/wk antigen	0.32 (0.03)	0.32 (0.03)	0.31 (0.03)	0.31 (0.03)
2.3 times/wk antigen, staff only	0.35 (0.04)	0.34 (0.04)	0.33 (0.04)	0.33 (0.04)
Daily antigen	0.29 (0.03)	0.30 (0.03)	0.29 (0.03)	0.30 (0.03)
Daily antigen, staff only	0.31 (0.03)	0.32 (0.03)	0.31 (0.03)	0.31 (0.04)
Weekly PCR	0.38 (0.05)	0.37 (0.04)	0.36 (0.04)	0.35 (0.04)
2.3 times/wk PCR	0.33 (0.04)	0.33 (0.04)	0.33 (0.04)	0.33 (0.03)
Daily PCR	0.31 (0.03)	0.30 (0.03)	0.30 (0.03)	0.31 (0.03)
**Total (residents and staff with nursing home, or community origin of infection)**				
None	0.41 (0.06)	0.40 (0.05)	0.36 (0.05)	0.38 (0.06)
Weekly antigen	0.28 (0.05)	0.28 (0.05)	0.28 (0.04)	0.28 (0.04)
Weekly antigen, staff only	0.30 (0.05)	0.29 (0.04)	0.28 (0.04)	0.28 (0.03)
2.3 times/wk antigen	0.24 (0.04)	0.24 (0.04)	0.24 (0.04)	0.23 (0.03)
2.3 times/wk antigen, staff only	0.26 (0.04)	0.25 (0.04)	0.25 (0.04)	0.24 (0.04)
Daily antigen	0.21 (0.03)	0.21 (0.03)	0.21 (0.03)	0.21 (0.03)
Daily antigen, staff only	0.22 (0.03)	0.22 (0.03)	0.22 (0.03)	0.22 (0.03)
Weekly PCR	0.32 (0.06)	0.30 (0.04)	0.29 (0.04)	0.29 (0.04)
2.3 times/wk PCR	0.26 (0.04)	0.26 (0.05)	0.26 (0.04)	0.26 (0.04)
Daily PCR	0.23 (0.03)	0.23 (0.03)	0.23 (0.03)	0.24 (0.03)

Abbreviation: PCR, polymerase chain reaction.

In the absence of testing, the estimated effectiveness associated with the immunity-based staffing intervention compared with no intervention was 19% for residents, 10% for staff, and 12% overall. The estimated effectiveness associated with this intervention was lower when testing frequency increased. Combining the resident cohorting and immunity-based staffing interventions was not associated with higher estimated effectiveness than the immunity-based staffing intervention alone (eFigure 2 in the Supplement) but again decreased the amount of PPE required. Results of each intervention and testing scenario were qualitatively consistent across simulations (eFigure 3 in the Supplement).

In the sensitivity analysis reflecting either lower PPE efficacy or imperfect use or absence of sufficient PPE, such that protection was 25% rather than 95%, the contact-targeted interventions were still associated with protecting residents in the absence of testing or when testing was only conducted weekly. However, lower PPE efficacy was associated with a higher cumulative incidence proportion for staff and residents compared with baseline, and the immunity-based staffing intervention was no longer associated with reduced transmission in most testing scenarios (eFigure 4 in the Supplement). We conducted 2 sensitivity analyses that increased the force of infection, one by doubling β, the probability of infection given contact (eFigure 5 and eFigure 6 in the Supplement), and the other by removing the relationship between viral load and infectiousness, such that individuals with viral load greater than 0 copies/mL were fully infectious (eFigure 7 in the Supplement). In these scenarios, the contact-targeted interventions were associated with higher estimated effectiveness for residents and staff for all testing strategies except daily testing. In the scenario with increased β, the immunity-based staffing intervention was associated with the largest estimated effectiveness, reducing mean cumulative incidence proportion by 25% for residents, 11% for staff, and 18% overall in the no testing scenario.

**Figure 2.**
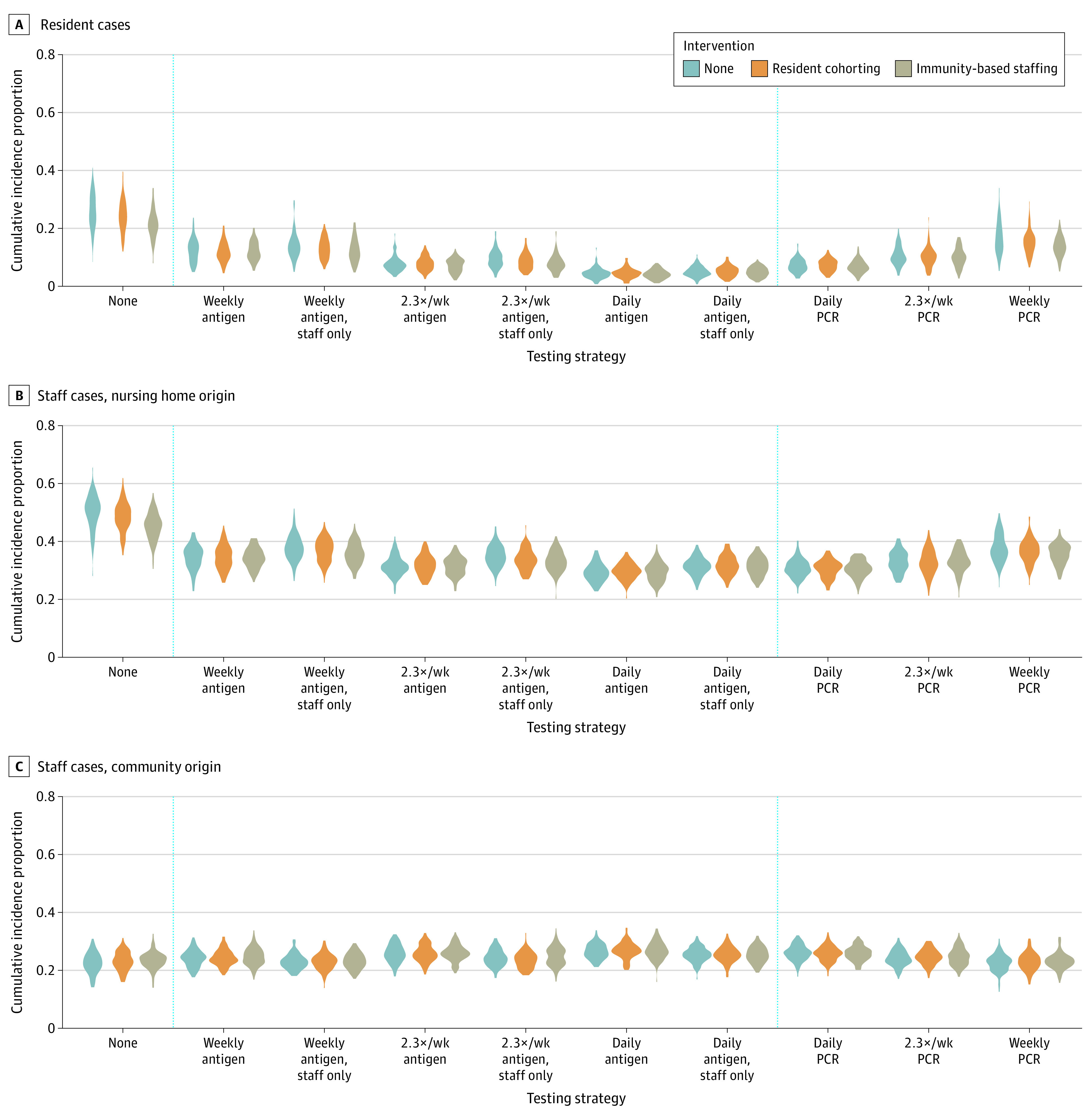
Cumulative Incidence Proportion at 3 Months From First SARS-CoV-2 Introduction Under Different Cohorting, Staffing, and Testing Interventions Violin plots show simulation results from 100 stochastic simulations. Cases among staff are split out to distinguish between cases arising in the community and those that are a result of transmission within the nursing home. PCR indicates polymerase chain reaction.

The frequency and type of testing was associated with a larger estimated impact on the size of outbreaks than the contact-targeted interventions (Figure 2). We modeled daily, every 3 days, or weekly testing, either of both staff and residents (PCR and antigen) or of staff only (antigen only). In all models, increasing the frequency of testing increased the effectiveness of testing. We calculated that antigen testing had a mean (SD) sensitivity for infection of 41% (16%) compared with 66% (13%) for PCR testing. However, the mean (SD) sensitivity of antigen tests for infectiousness was 74% (14%). Because of the infectiousness cutoff, PCR test sensitivity for infectiousness is 100%. Despite imperfect sensitivity, antigen tests were associated with greater reductions in transmission than PCR tests at the same frequency owing to the faster turnaround time. Daily antigen testing of everyone or of staff only was associated with the greatest estimated effectiveness, reducing mean cumulative incidence proportion by 49% for testing everyone and 46% for testing staff only in the absence of contact-targeted interventions compared with no testing. In contrast, daily PCR testing was associated with an estimated effectiveness of 44%. These testing interventions were associated with particularly high estimated effectiveness among residents, with 85% reduction from daily antigen testing, 81% from daily antigen testing among staff only, and 73% from daily PCR testing. When conducted weekly, antigen testing was associated with an estimated effectiveness of 32%, and PCR testing was associated with an estimated effectiveness of 22%. Under more effective testing strategies, ongoing transmission within the nursing home was effectively prevented, and cumulative incidence primarily reflected continued community introductions (eFigure 8 in the Supplement).

When antigen tests had lower sensitivity (mean [SD] 25% [18%] for infectiousness when LOD = 10^7^) (eFigure 2 in the Supplement), daily antigen testing was associated with lower estimated effectiveness than daily PCR testing, which reduced overall cumulative incidence proportion by 41% for antigen testing and 44% for PCR testing. However, with the baseline sensitivity, even with a 1-day turnaround time (vs 2 days at baseline), weekly PCR testing was associated with lower effectiveness (29%) than weekly antigen testing (32%) (eFigure 2 in the Supplement). In simulations with imperfect specificity and a 2-day lag for confirmatory testing, we saw no change in the overall trends; however, cumulative incidence was higher (eFigure 9 in the Supplement). Imperfect specificity increased the number of temporary staff required, especially at greater testing frequencies, creating more opportunities for importations and an additional burden on nursing home operations.

**Figure 3.**
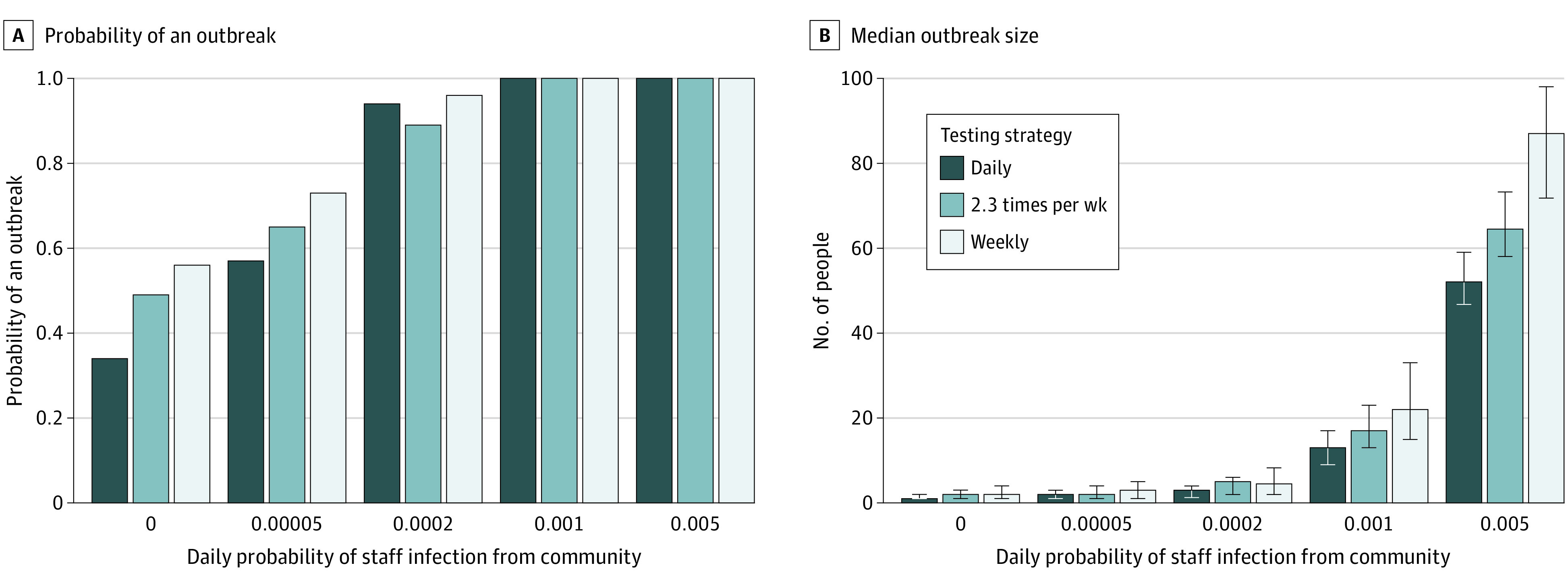
Probability and Size of an Outbreak Under Varying Community Prevalence Settings 0 indicates a single initial case but no additional community introductions (ie, no infection from the community). Error bars indicate interquartile ranges.

Staff community infection risk, which depends on community prevalence, was associated with outbreak size and intervention estimated effectiveness (Figure 3; eFigure 10 in the Supplement). When community infection risk was lower, the contact-targeted interventions were not associated with any change in the mean cumulative incidence proportion among residents. In addition, reducing the frequency of testing was associated with a smaller change in final outbreak size. Weekly antigen testing was associated with smaller reductions in transmission (50%) than daily antigen testing (69%), and PCR tests performed 2.3 times per week were associated with smaller reductions in transmission (56%) than daily PCR (63%). However, since the mean cumulative incidence proportion was low, there were minimal differences in absolute values—suggesting that in lower-prevalence settings, less frequent testing may suffice. At higher community prevalence, outbreaks (ie, any onward transmission) always occurred, but outbreak size depended on testing frequency and interventions (Figure 3; eFigure 11 in the Supplement). At low community prevalence settings, the probability of an outbreak depended on the testing frequency, but once an outbreak occurred, testing was associated with a smaller effect on outbreak size. However, in simulations with a high β and a low introduction probability, both testing and contact-targeted interventions were associated with decreased transmission compared with baseline, and there was greater variation in cumulative incidence proportion (eFigure 6 in the Supplement).

Increasing contact rates, either through nonroommate interactions among residents (eFigure 12 in the Supplement) or decreasing the staff-to-resident ratio, thus increasing the number of resident contacts per staff member (eFigure 13 in the Supplement), was associated with increased transmission within the nursing home compared with baseline. In the scenario with increased resident-resident interactions, the mean (SD) cumulative incidence proportion in the absence of interventions and testing was 0.29 (0.06) for residents, 0.51 (0.06) for staff infected in the nursing home, and 0.43 (0.06) for total nursing home infections, compared with baseline, with mean (SD) incidences of 0.26 (0.06) for residents, 0.50 (0.06) for staff infected in the nursing home, and 0.41 (0.06) for total nursing home infections. In the scenario with decreased staff, the mean (SD) cumulative incidence proportions were 0.33 (0.07) for residents, 0.62 (0.08) for staff infected in the nursing home, and 0.42 (0.07) for total nursing home infections. Although the mean cumulative incidence proportion was sensitive to these parameters, the relative transmission outcomes of testing and contact-targeted interventions were similar.

## Discussion

The findings of this decision analytical modeling study suggest that increasing the frequency of screening testing of all residents and staff, or even staff alone, in nursing homes has the potential to reduce outbreaks. Our results provide further support to other models’ findings of the benefit of frequent and rapid testing^23^ and that faster results should be prioritized over high sensitivity,^14,24^ even in small congregate settings, like nursing homes. While rapid antigen testing generally performs better than PCR testing, which is more sensitive but has a longer turnaround and may be prohibitively expensive, we found that when the limit of detection was very high for antigen testing, this did not hold. This suggests there may be a sensitivity threshold past which antigen testing becomes ineffective.

The staffing intervention we propose may further reduce spread among residents at little or no additional cost and becomes particularly important when frequent testing is not feasible. It may have the additional advantage of being more straightforward to implement than our cohorting intervention, which involves moving residents. The staffing intervention was associated with reduced risk of staff infecting residents who were susceptible by prioritizing recovered staff to work with them, lowering the effective reproduction number in the non–COVID-19 cohort. This intervention hinges on the assumption that recovered staff are protected against reinfection. Access to appropriate PPE and training on proper use are required for it to be both feasible and ethical. Because we assume a constant rate of community introduction, the change in staff infections within the nursing home associated with contact-targeted interventions is often compensated for by infections acquired in the community.

Our results suggest that, given limited resources, community prevalence could guide the choice of interventions. When prevalence was lower, contact-targeted interventions were associated with smaller reductions in cumulative incidence proportion, because most individuals’ contacts were susceptible. We also found that increased testing frequency was associated with smaller reductions in infection incidence, suggesting that when community prevalence is low and testing resources are limited, nursing homes could consider using weekly testing until an outbreak is detected. Models of similar settings have also found the frequency of testing required to control outbreaks can be dependent on community prevalence.^25^ When outbreaks were very large, we found that the staffing intervention was associated with greater reductions in cumulative incidence among residents and staff. Expanding this model to explore the timings and thresholds for implementing reactive strategies in nursing homes without detected outbreaks^26^ is an important area for future work.

### Limitations

This study has some limitations. In developing and analyzing our model, we have made several simplifying assumptions. We assume no spillover between the COVID-19 and non–COVID-19 cohorts. In settings with understaffing, this assumption may be violated owing to compounding effects associated with outbreak-related stressors. As a conservative assumption, we assume no difference in viral load dynamics between symptomatic and asymptomatic individuals and ignore the persistent low viral loads after individuals are no longer infectious^19^: because we are looking at frequent testing strategies, we do not expect these viral load dynamics to affect our results. While we incorporate varying transmission risk by type of contact (resident-staff, resident-resident, and staff-staff), we do not explicitly model different staff roles, which may have different levels of risk. We made this simplification in order to make the results of this model more generalizable, as nursing home staff structure varies widely.

We also assume that all individuals with symptoms are infected with SARS-CoV-2 and do not model symptoms from other diseases. Given low rates of other infectious diseases, such as flu, due in part to non-pharmaceutical interventions, we think this is a reasonable assumption.^27^ While we incorporate length of stay data for residents and temporary staff for staff who are isolating at home, we do not model staff turnover. Recent research suggests nursing home staff turnover may be very high.^28^ The impact of this assumption for the results of our model would depend heavily on the levels of immunity in new staff: new staff could come from other nursing homes that have experienced outbreaks or from the general community, which would likely have lower seroprevalence. In addition, available data are from before the COVID-19 pandemic, and there may be large heterogeneity in how the pandemic affected staff employment trends. In an effort to minimize the implications of this assumption on our results, we limit our analysis to only 3 months.

## Conclusions

This decision analytical modeling study found that simple interventions involving testing and staffing were associated with greatly reducing the burden of SARS-CoV-2 in nursing homes. In practice, the choice of interventions would depend on the resources available, which include testing, PPE, and staff time. Our model further underscores the importance of rapid screening testing.

## Notes

Supplement. **eTable 1.** Parameters **eTable 2. **Testing Interventions **eFigure 1.** Number of Staff-Days in the COVID Cohort by Testing Strategy **eFigure 2.** Additional Intervention Strategies **eFigure 3.** Individual Simulations **eFigure 4.** Lower PPE Effectiveness **eFigure 5.** Higher Force of Infection With Baseline Community Prevalence **eFigure 6.** Higher Force of Infection With Lower Community Prevalence **eFigure 7.** Binary Infectiousness, Unrelated to Viral Load **eFigure 8.** Mean Cumulative Incidence by Testing Scenario **eFigure 9.** Imperfect Specificity **eFigure 10.** Lower Daily Probability of Staff Infection From the Community **eFigure 11.** Cumulative Incidence by Daily Probability of Infection From the Community and Testing Strategy **eFigure 12.** Higher Resident-Resident Contacts **eFigure 13.** Lower Staff-Resident Ratio eReferences
