# Supplementary material for: Estimation of Transmission of COVID-19 in Simulated Nursing Homes With Frequent Testing and Immunity-Based Staffing
Source: JAMA Netw Open. 2021 May 14;4(5):e2110071. doi: 10.1001/jamanetworkopen.2021.10071 (PMC8122229; doi:10.1001/jamanetworkopen.2021.10071)
Supplement: Supplement. — eTable 1. Parameters eTable 2. Testing Interventions eFigure 1. Number of Staff-Days in the COVID Cohort by Testing Strategy eFigure 2. Additional Intervention Strategies eFigure 3. Individual Simulations eFigure 4. Lower PPE Effectiveness eFigure 5. Higher Force of Infection With Baseline Community Prevalence eFigure 6. Higher Force of Infection With Lower Community Prevalence eFigure 7. Binary Infectiousness, Unrelated to Viral Load eFigure 8. Mean Cumulative Incidence by Testing Scenario eFigure 9. Imperfect Specificity eFigure 10. Lower Daily Probability of Staff Infection From the Community eFigure 11. Cumulative Incidence by Daily Probability of Infection From the Community and Testing Strategy eFigure 12. Higher Resident-Resident Contacts eFigure 13. Lower Staff-Resident Ratio eReferences [file jamanetwopen-e2110071-s001.pdf]

Supplemental Online Content

Holmdahl I, Kahn R, Hay JA, Buckee CO, Mina MJ. Estimation of transmission of COVID-19 in simulated nursing homes with frequent testing and immunity-based staffing. *JAMA Netw Open*. 2021;4(5):e2110071. doi:10.1001/jamanetworkopen.2021.10071

eTable 1. Parameters
eTable 2. Testing Interventions
eFigure 1. Number of Staff-Days in the COVID Cohort by Testing Strategy
eFigure 2. Additional Intervention Strategies
eFigure 3. Individual Simulations
eFigure 4. Lower PPE Effectiveness
eFigure 5. Higher Force of Infection With Baseline Community Prevalence
eFigure 6. Higher Force of Infection With Lower Community Prevalence
eFigure 7. Binary Infectiousness, Unrelated to Viral Load
eFigure 8. Mean Cumulative Incidence by Testing Scenario
eFigure 9. Imperfect Specificity
eFigure 10. Lower Daily Probability of Staff Infection From the Community
eFigure 11. Cumulative Incidence by Daily Probability of Infection From the Community and Testing Strategy
eFigure 12. Higher Resident-Resident Contacts
eFigure 13. Lower Staff-Resident Ratio
eReferences

This supplemental material has been provided by the authors to give readers additional information about their work.

© 2021 Holmdahl I et al. *JAMA Network Open.*

eTable 1. Parameters

**Parameter**	**Values****
Number of residents	100[1]
Number of staff	100, 50[1]
Probability of infection per infectious contact	0.02, 0.04
Latent period (days)	3-5[2]
Time in infectious compartment (days)*	14
Daily probability of infection from the community	0.005, 0.001, 0.0002, 0.00005, 0
Daily contacts staff-staff	2[1]
Daily contacts residents - staff	6[1]
Daily contacts staff - residents	6, 12[1]
Daily contacts residents - residents (non roommates)	0, 2[1]
Proportion of staff asymptomatic	0.4[3]
Proportion of residents asymptomatic	0.2[4,5]
Duration of presymptomatic transmission (days)	2[2,6]
Reduction in force of infection per contact from PPE	95%, 25%[7]
Proportion of temporary healthcare workers recovered upon	0.2

© 2021 Holmdahl I et al. *JAMA Network Open.*

entry into nursing home	
Baseline mortality or discharge (daily)	1/1000, 1/60
COVID mortality or discharge (daily)	2/100
Mean peak viral load (copies/mL)	10^8[8]
Limit of detection - rapid antigen test (copies/mL)	10^5, 10^7[9-12]
Limit of detection - PCR (copies/mL)	10^3[9-13]
Antigen test specificity	1, 0.995
Viral load threshold for infectiousness (copies/mL)	10^4
Viral load threshold for high infectiousness (copies/mL)	10^7
Turn around time - antigen test (days)	Same day
Turn around time - PCR test (days)	2, 7
Days until cohorting interventions begin	21
Minimum length of stay (days)	7

*Duration of infectiousness depends on viral load trajectory with a maximum of 14 days. In one example simulation, the median days of infectiousness is 6.3, resulting in an R_0 of 3.4 for residents and 2.5 for staff.

**If no reference is cited, parameters are assumed.

© 2021 Holmdahl I et al. *JAMA Network Open.*

eTable 2. Additional Testing Interventions

Mix	Daily antigen	Weekly PCR	Limit of detection	Turnaround time, d
Antigen	3.5×/wk	3.5×/wk	10^5	<1
Antigen	3.5×/wk	NA	10^5	<1
Antigen	Daily	Daily	10^7	<1
PCR	Weekly	Weekly	10^3	7
PCR	Weekly	Weekly	10^3	1

© 2021 Holmdahl I et al. *JAMA Network Open.*

eFigure 1. Number of Staff-Days in the COVID Cohort by Testing Strategy

The number of staff-days in the COVID cohort varies by intervention within each testing strategy. When resident cohorting is used, either alone or with immunity-based staffing, the number of staff-days in the COVID cohort is lower over the course of the outbreak.

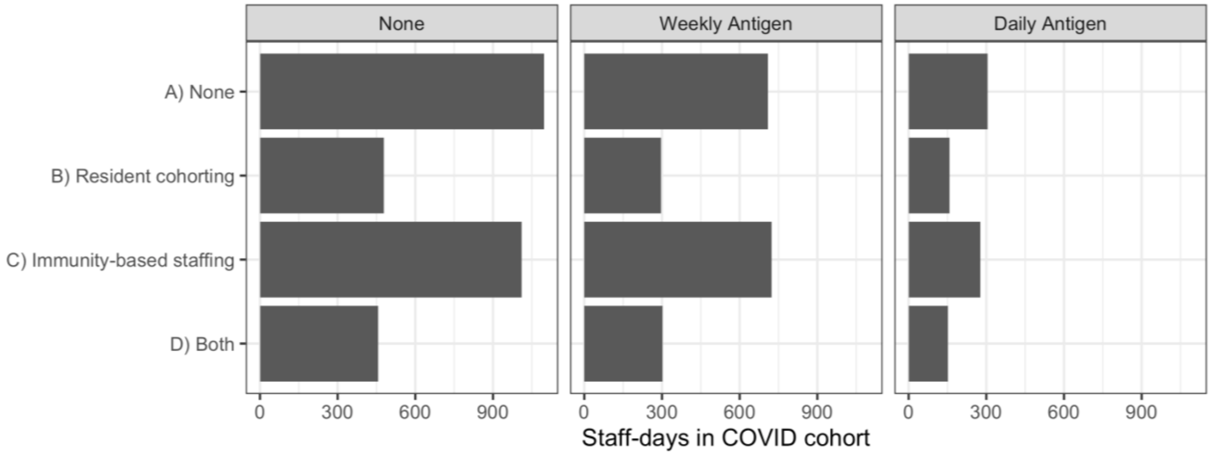

© 2021 Holmdahl I et al. *JAMA Network Open.*

eFigure 2. Additional Intervention Strategies

Cumulative incidence at 3 months from first SARS-CoV-2 introduction under an extended set of testing and intervention strategies. Testing strategies included here but not in the main text (Figure 2) are results for testing every other day (3.5x/week) as well as sensitivity analyses. A combination of the resident cohorting and immunity-based staffing strategies is also shown in purple.

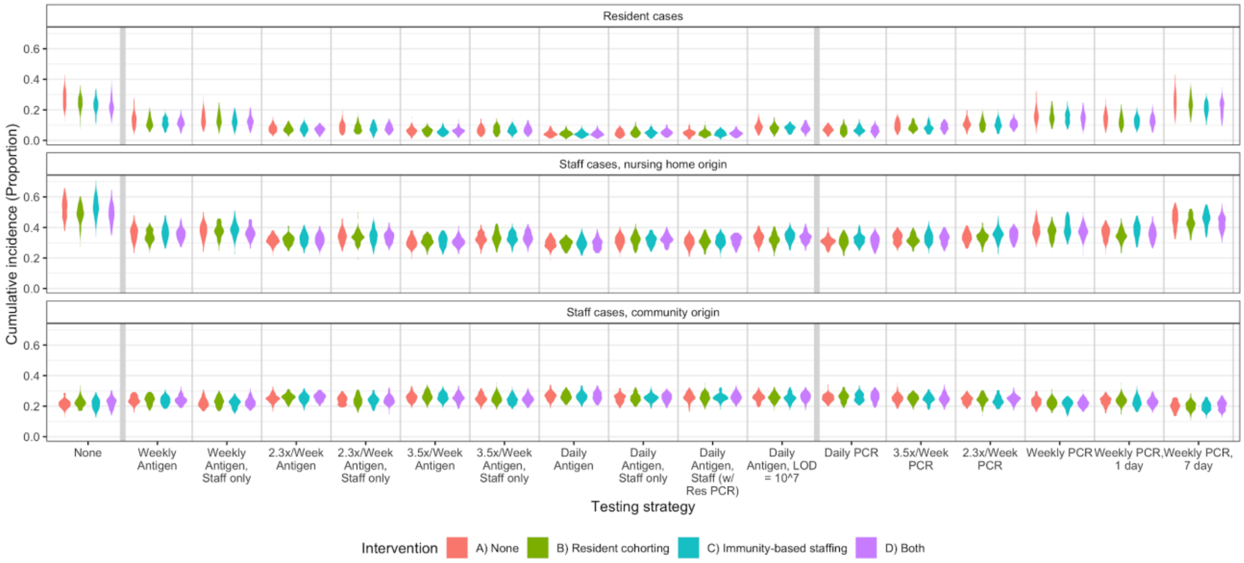

© 2021 Holmdahl I et al. *JAMA Network Open.*

eFigure 3. Individual Simulations

Plotting individual simulation trajectories show that outbreak dynamics remain relatively consistent across simulations. Solid lines are residents, and dashed lines are staff.

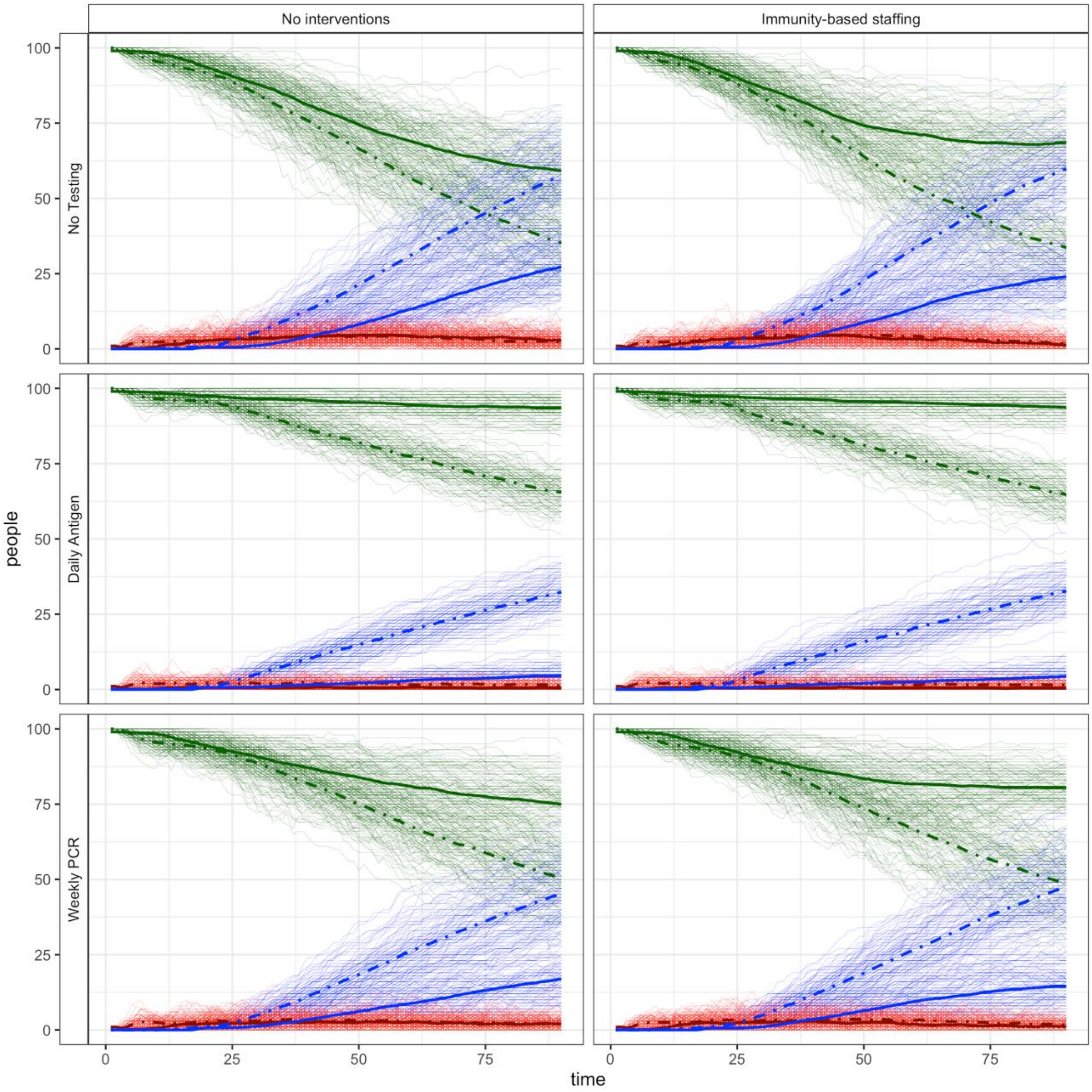

© 2021 Holmdahl I et al. *JAMA Network Open.*

eFigure 4. Lower PPE Effectiveness

When PPE effectiveness is lower, intervention and testing effectiveness follows the same general trends, but with slightly higher cumulative incidence and no effectiveness of the immunity-based staffing intervention among staff.

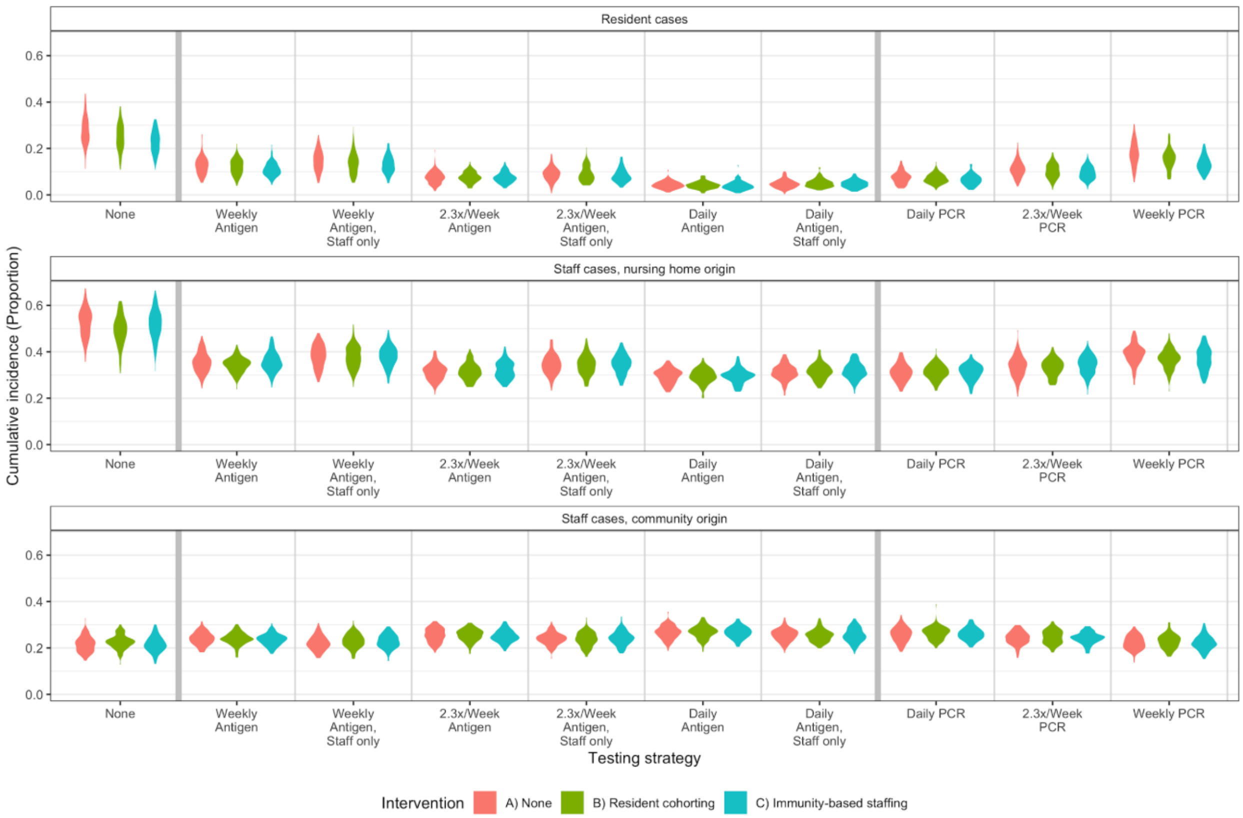

© 2021 Holmdahl I et al. *JAMA Network Open.*

eFigure 5. Higher Force of Infection With Baseline Community Prevalence

When the force of infection is higher, both testing and contact-targeted interventions have a larger effect than in the baseline scenario.

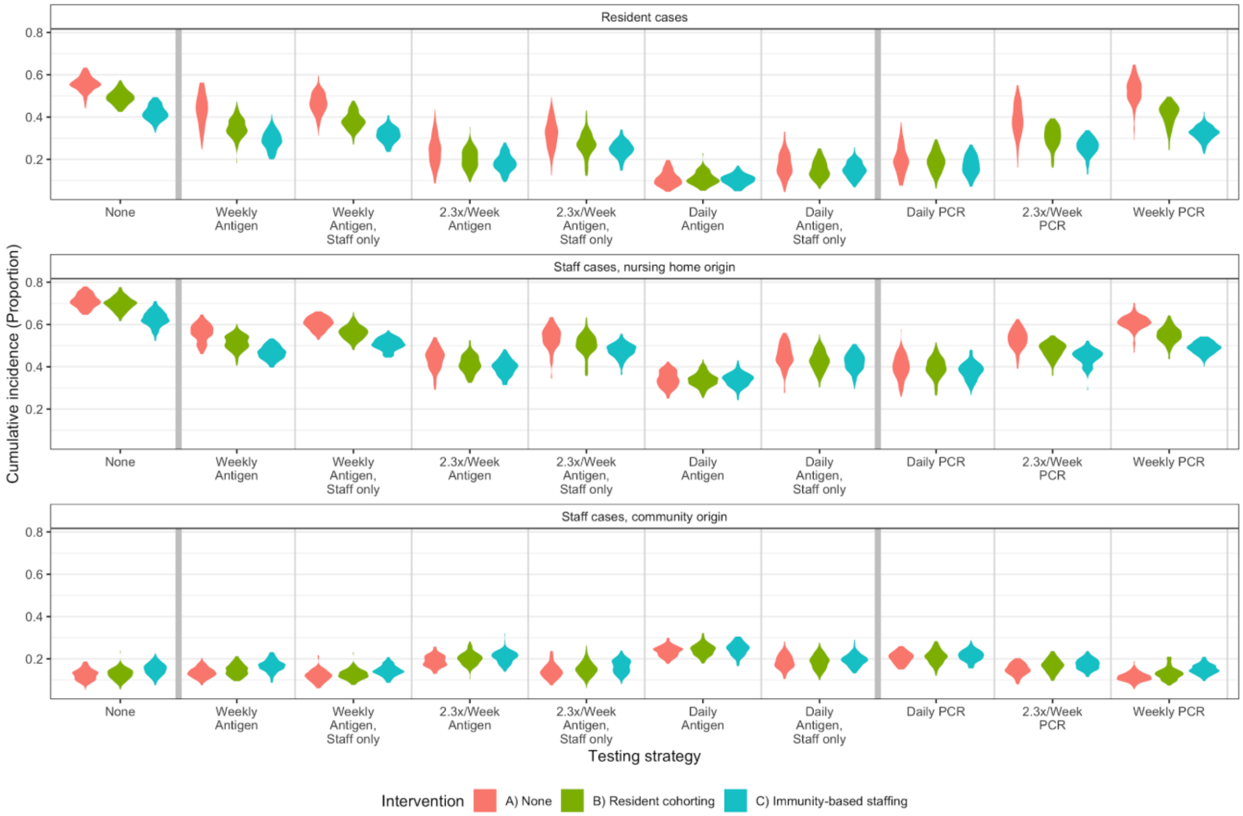

© 2021 Holmdahl I et al. *JAMA Network Open.*

eFigure 6. Higher Force of Infection With Lower Community Prevalence

When the force of infection is higher and community prevalence is lower, both testing and contact-targeted interventions have a larger effect than in the baseline scenario, and there is greater variation in cumulative incidence proportion.

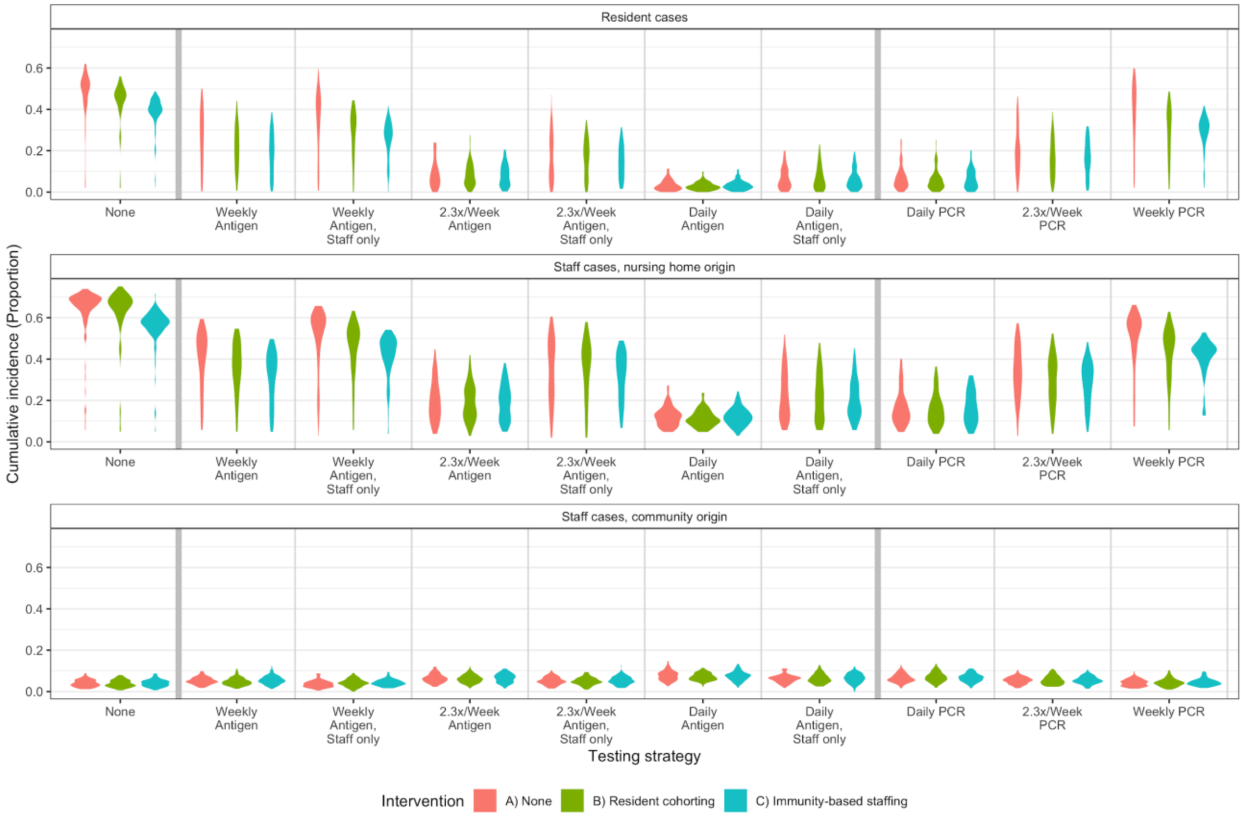

© 2021 Holmdahl I et al. *JAMA Network Open.*

eFigure 7. Binary Infectiousness, Unrelated to Viral Load

When infectiousness begins as soon as VL is greater than 0, and is not related to VL, the contact-targeted interventions are more effective than in the baseline scenario.

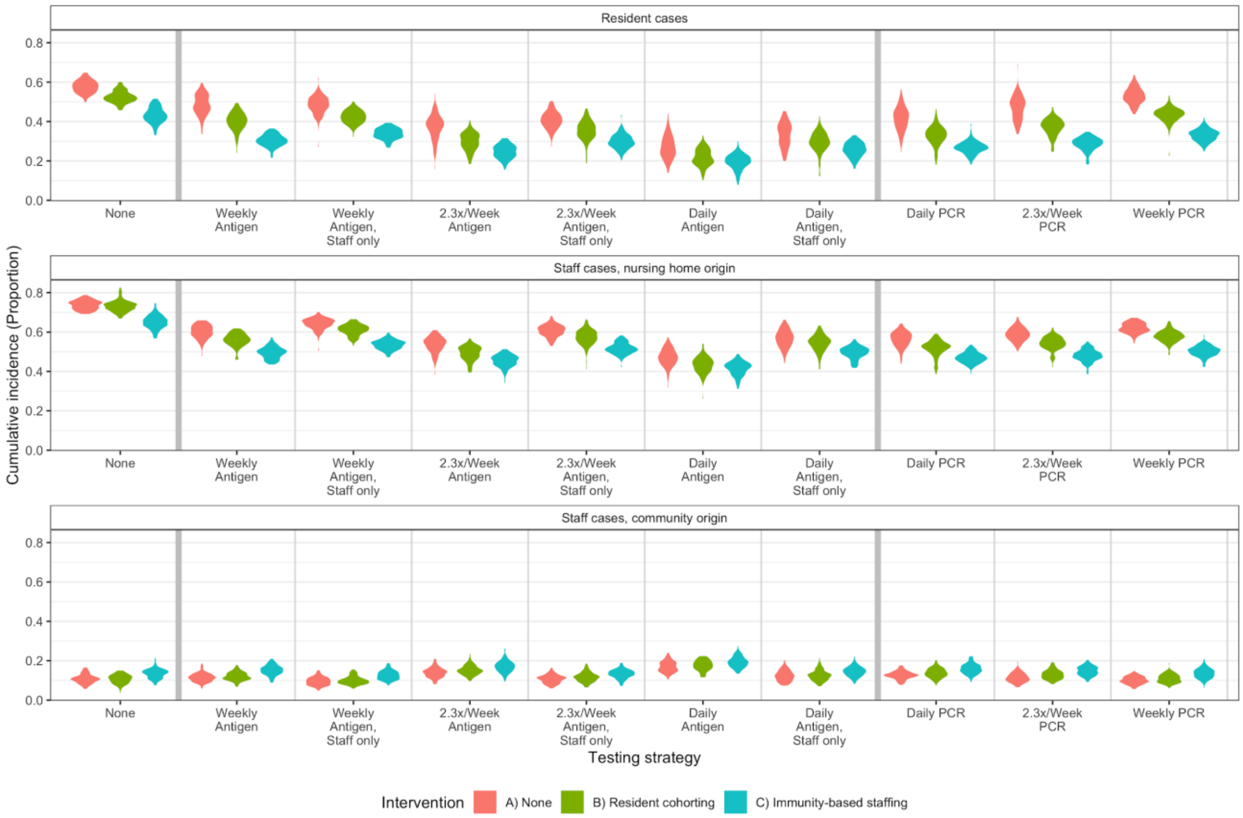

© 2021 Holmdahl I et al. *JAMA Network Open.*

eFigure 8. Mean Cumulative Incidence by Testing Scenario

Mean cumulative incidence proportion over 3 months of simulation in each baseline testing scenario. Solid lines indicate cases in residents; dotted lines indicate cases in staff that were introduced from the community, and dashed lines indicate cases in staff that occurred inside the nursing home.

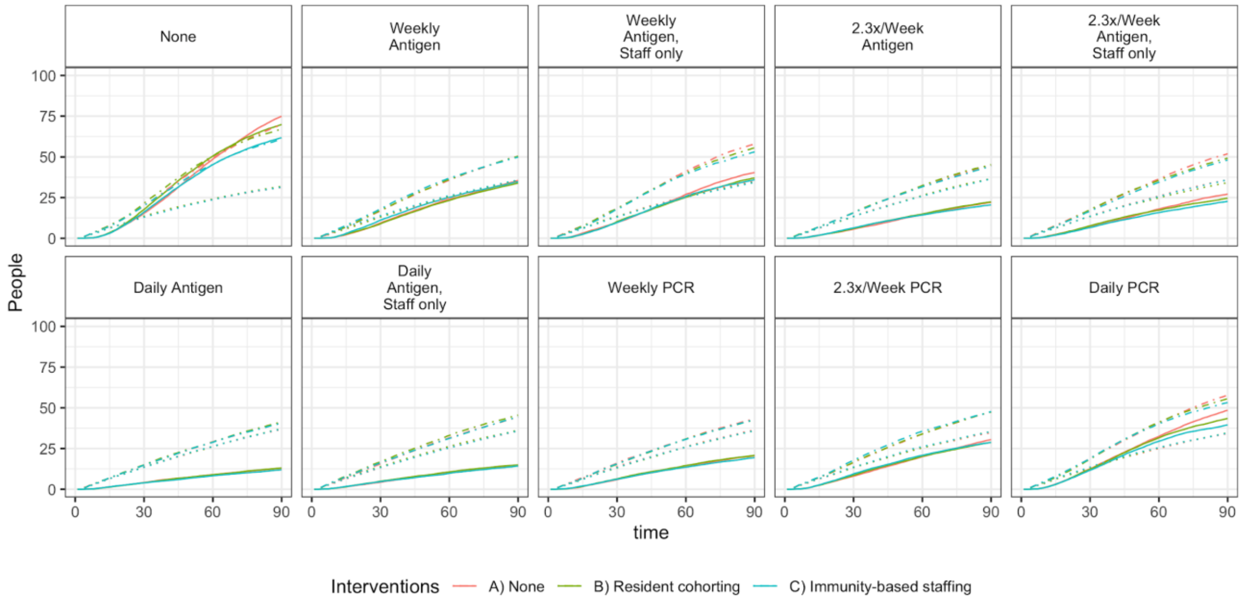

© 2021 Holmdahl I et al. *JAMA Network Open.*

eFigure 9. Imperfect Specificity

When antigen test specificity is imperfect, intervention and testing effectiveness follows the same general trends as when specificity is 100%. However, additional temporary workers are required.

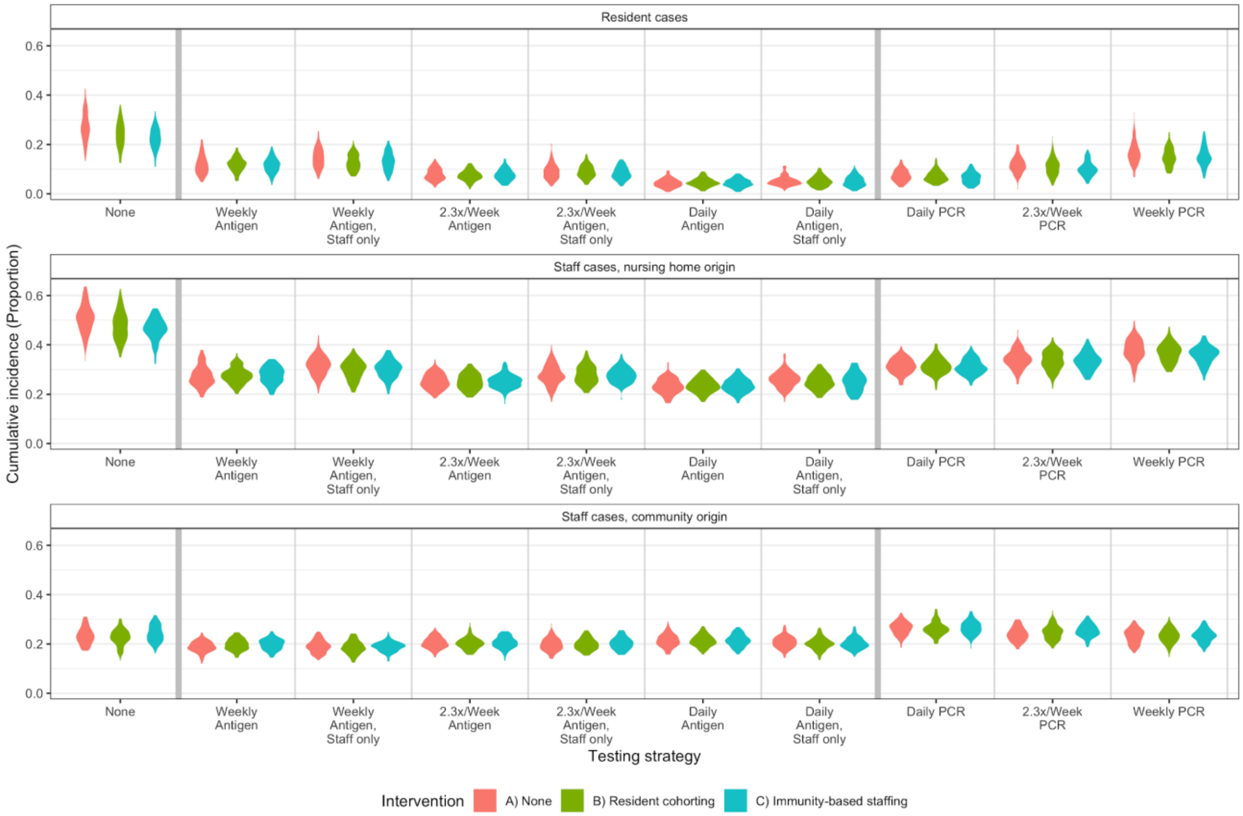

© 2021 Holmdahl I et al. *JAMA Network Open.*

eFigure 10. Lower Daily Probability of Staff Infection From the Community

When daily probability of staff infection is lower, contact-targeted interventions and testing frequency do not affect cumulative incidence as much as at higher probability of infection.

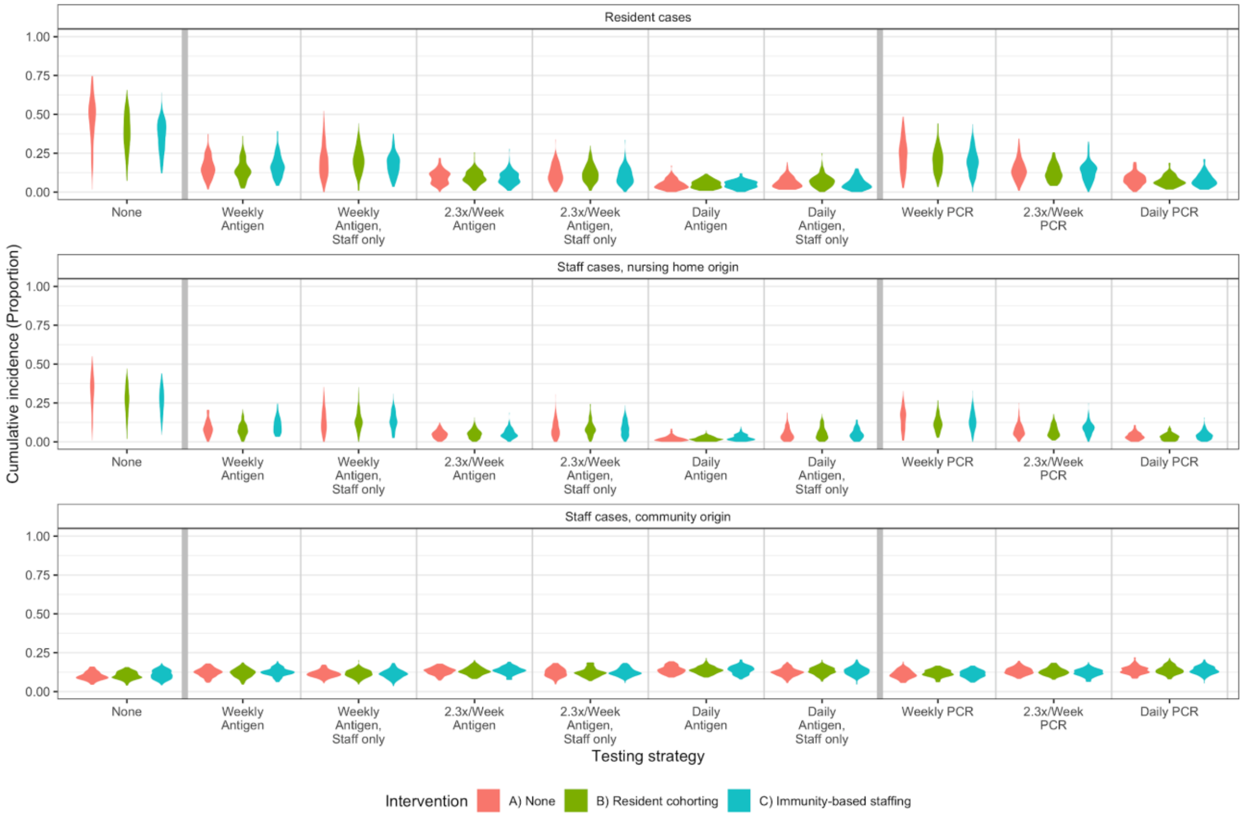

© 2021 Holmdahl I et al. *JAMA Network Open.*

eFigure 11. Cumulative Incidence by Daily Probability of Infection From the Community and Testing Strategy

Cumulative incidence (100 simulations) by daily probability of infection from the community (columns) and testing strategy (rows), under no contact-targeted intervention. This excludes staff members infected from the community. Although they are less common at lower probabilities of infection from the community (or when seeded with only one case, as in the far left column), larger outbreaks do occur when testing is less frequent.

© 2021 Holmdahl I et al. *JAMA Network Open.*

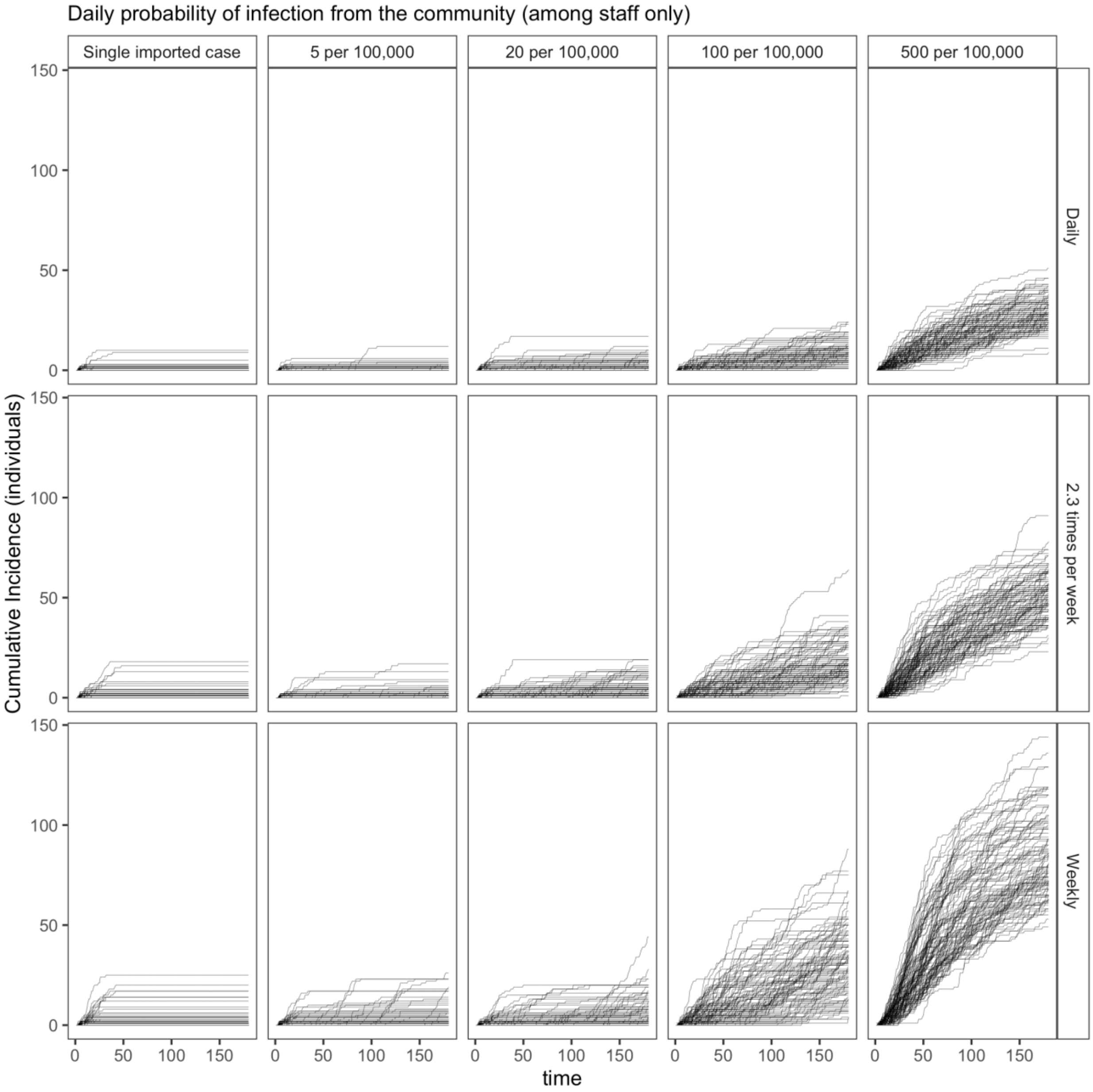

© 2021 Holmdahl I et al. *JAMA Network Open.*

eFigure 12. Higher Resident-Resident Contacts

When the number of contacts between residents is increased beyond roommates only, the relative effectiveness of interventions and testing does not change.

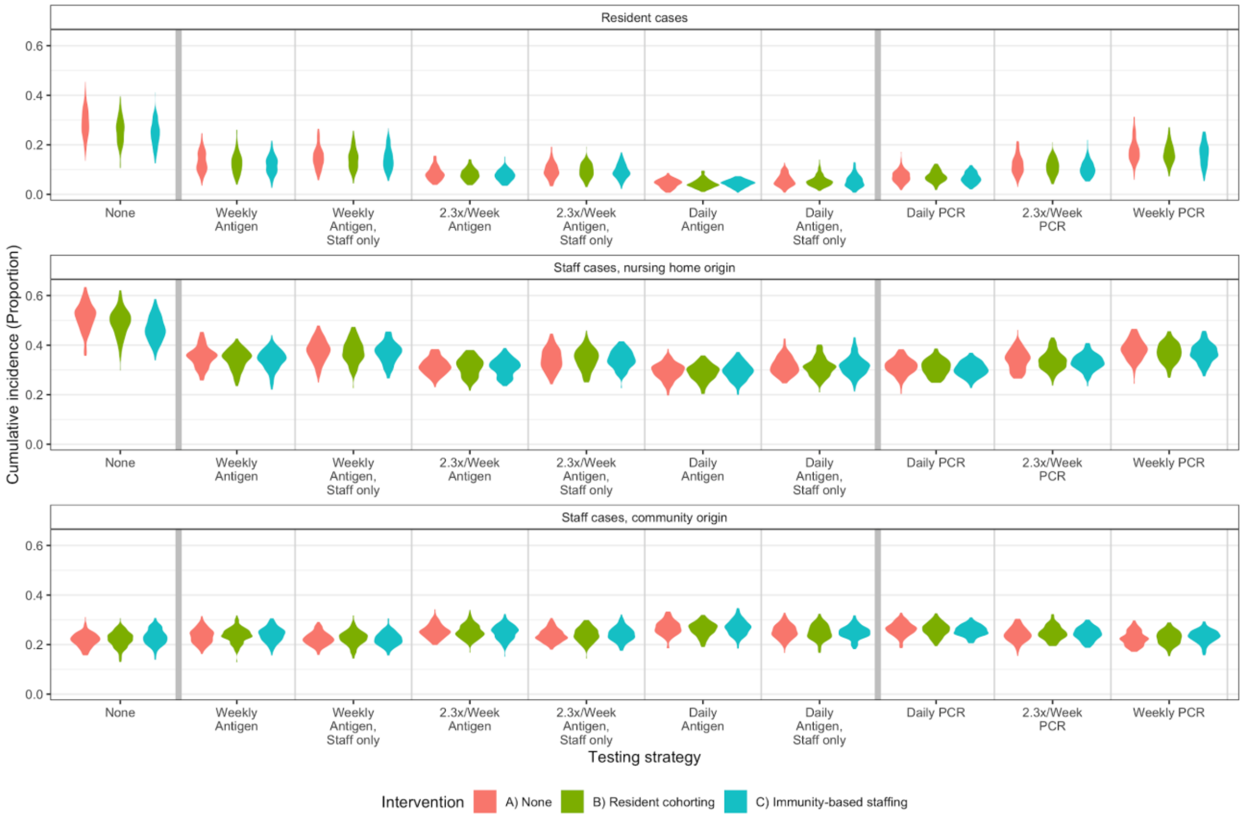

© 2021 Holmdahl I et al. *JAMA Network Open.*

eFigure 13. Lower Staff-Resident Ratio

When the ratio of staff to residents is decreased, intervention effectiveness does not change.

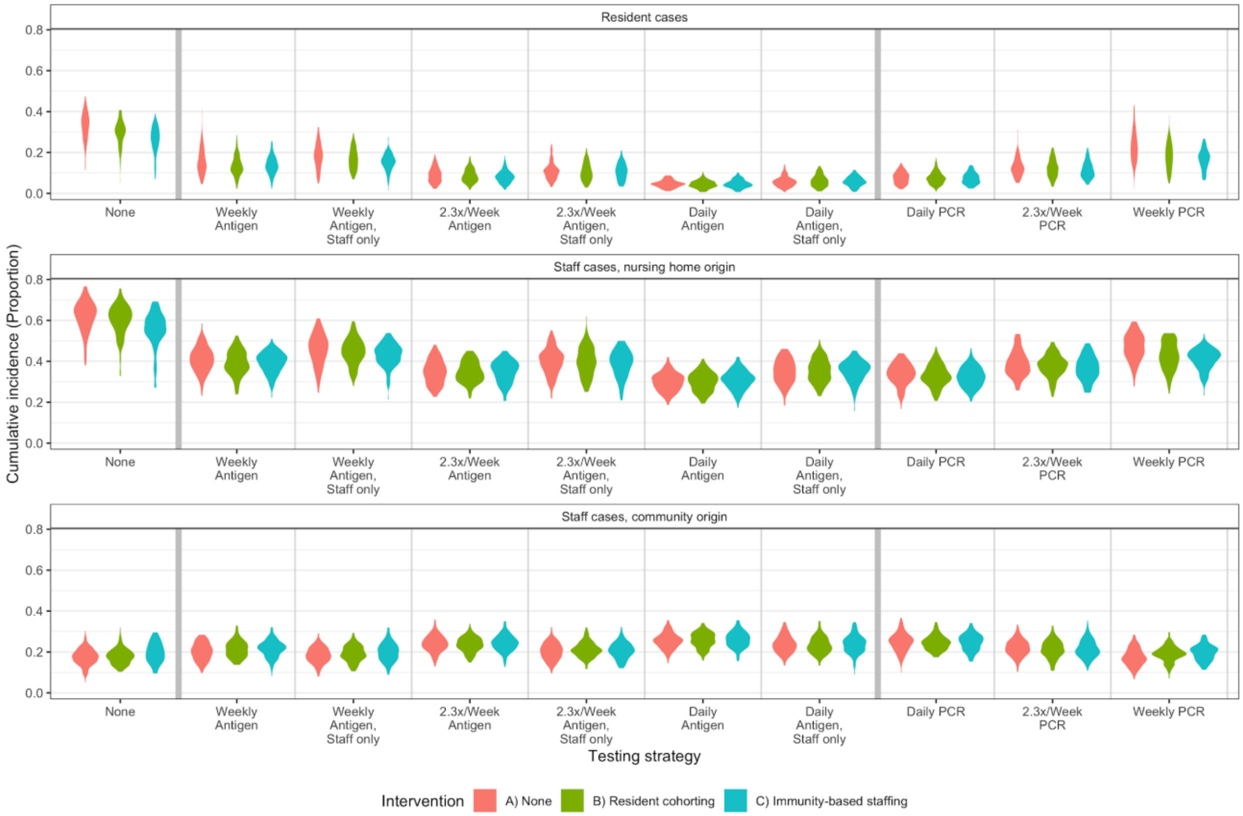

© 2021 Holmdahl I et al. *JAMA Network Open.*

eReferences

1. Anglo R. Chief Clinical Officer, Chelsea Jewish Life Care, Personal communication, June 10, 2020.
2. Bar-On YM, Flamholz A, Phillips R, Milo R. SARS-CoV-2 (COVID-19) by the numbers. *Elife*. 2020;9:e57309. Medline:32228860 doi:10.7554/eLife.57309
3. Oran DP, Topol EJ. Prevalence of asymptomatic SARS-CoV-2 infection: a narrative review. *Ann Intern Med*. 2020;173(5):362-367. Medline:32491919 doi:10.7326/M20-3012
4. Centers for Disease Control and Prevention. Coronavirus disease 2019 (COVID-19). September 11, 2020. Accessed September 14, 2020. https://www.cdc.gov/coronavirus/2019-ncov/hcp/planning-scenarios.html
5. Byambasuren O, Cardona M, Bell K, Clark J, McLaws M-L, Glasziou P. Estimating the extent of true asymptomatic COVID-19 and its potential for community transmission: systematic review and meta-analysis. *Off J Assoc Med Microbiol Infect Dis Can*. 2020;5(4):223-234. doi:10.3138/jammi-2020-0030
6. Li Q, Guan X, Wu P, et al. Early transmission dynamics in Wuhan, China, of novel coronavirus-infected pneumonia. *N Engl J Med*. 2020;382(13):1199-1207. Medline:31995857 doi:10.1056/NEJMoa2001316
7. Livingston E, Desai A, Berkwits M. Sourcing personal protective equipment during the COVID-19 pandemic. *JAMA*. 2020;323(19):1912-1914. Medline:32221579 doi:10.1001/jama.2020.5317
8. Wölfel R, Corman VM, Guggemos W, et al. Virological assessment of hospitalized patients with COVID-2019. *Nature*. 2020;581(7809):465-469. Medline:32235945 doi:10.1038/s41586-020-2196-x
9. Larremore DB, Wilder B, Lester E, et al. Test sensitivity is secondary to frequency and turnaround time for COVID-19 screening. *Sci Adv*. 2021;7(1):eabd5393. Medline:33219112 doi:10.1126/sciadv.abd5393
10. Butler DJ, Mozsary C, Meydan C, et al. Shotgun transcriptome and isothermal profiling of SARS-CoV-2 infection reveals unique host responses, viral diversification, and drug interactions. *bioRxiv*. Preprint posted online May 1, 2020. Accessed April 6, 2021. doi:10.1101/2020.04.20.048066
11. Dao Thi VL, Herbst K, Boerner K, et al. A colorimetric RT-LAMP assay and LAMP-sequencing for detecting SARS-CoV-2 RNA in clinical samples. *Sci Transl Med*. 2020;12(556):eabc7075. Medline:32719001 doi:10.1126/scitranslmed.abc7075
12. Meyerson NR, Yang Q, Clark SK, et al. A community-deployable SARS-CoV-2 screening test using raw saliva with 45 minutes sample-to-results turnaround. *medRxiv*. Preprint posted online July 17, 2020. Accessed April 6, 2021. doi:10.1101/2020.07.16.20150250
13. Vogels CBF, Brito AF, Wyllie AL, et al. Analytical sensitivity and efficiency comparisons of SARS-COV-2 qRT-PCR primer-probe sets. *medRxiv*. Preprint posed online April 1, 2020. Accessed April 6, 2021. doi:10.1101/2020.03.30.20048108

© 2021 Holmdahl I et al. *JAMA Network Open.*
